# ﻿Morphological and phylogenetic analyses reveal three new species of *Apiospora* in China

**DOI:** 10.3897/mycokeys.99.108384

**Published:** 2023-10-20

**Authors:** Shuji Li, Cheng Peng, Rong Yuan, Chengming Tian

**Affiliations:** 1 The Key Laboratory for Silviculture and Conservation of the Ministry of Education, Beijing Forestry University, Beijing 100083, China Beijing Forestry University Beijing China

**Keywords:** Apiosporaceae, Ascomycota, morphology, phylogeny, taxonomy

## Abstract

Species of *Apiospora* are distributed worldwide as endophytes, pathogens and saprobes. In this study, we analysed *Apiospora* strains isolated from diseased leaves in Yunnan Province and dead culms in Shaanxi Province, China and we identified fungal species based on multi-locus phylogeny of ITS, LSU, *tef1* and *tub2* genes, along with the morphological characters, host and ecological distribution. Analyses revealed three new species, namely *A.coryli***sp. nov.**, *A.lophatheri***sp. nov.** and *A.oenotherae***sp. nov.** and one known species *A.arundinis*. Illustrations and descriptions of the four taxa are provided, along with comparisons with closely-related taxa in the genus.

## ﻿Introduction

Species in *Apiospora* are distributed worldwide, primarily in temperate and tropical regions. These fungi can be found in various habitats, including soil, plant materials and insect exoskeletons (Pintos and Alvarado 2021). Many species of *Apiospora* are associated with plants as endophytic or saprophytic taxa and some can be important plant pathogens (Crous and Groenewald 2013; Wang et al. 2018; Kwon et al. 2021). In recent years, researchers have continuously discovered new *Apiospora* species in China (Wang et al. 2018; Senanayake et al. 2020, 2023; Feng et al. 2021; Liu et al. 2023).

*Apiospora*, the type genus of Apiosporaceae, was recognised and established by Saccardo (1875) with *A.montagnei* as the type species. For a long time, *Apiospora* was believed to be the sexual state of the genus *Arthrinium* (Ellis 1965; Samuels et al. 1981; Crous and Groenewald 2013). However, Ellis (1965) synonymised several other asexual genera with basauxic conidiogenesis under *Arthrinium*, such as *Papularia*, which was considered the asexual morph of *Apiospora* by von Höhnel (1919), Petrak (1925) and Hudson (1960, 1963). The asexual morph of *Apiospora* and *Arthrinium* are difficult to differentiate, based on morphology alone and the morphological relationships between *Arthrinium* and *Apiospora* have been hotly debated since Ellis (1965).

With the help of molecular phylogeny, *Apiospora* and *Arthrinium* were initially categorised in their own family Apiosporaceae (Hyde et al. 1998). Later, Crous and Groenewald (2013) considered that *Apiospora* was actually the sexual form of *Arthrinium* and both genera aligned to form a monophyletic clade. Following the principle of one fungi, one name policy (Hawksworth et al. 2011), the older name *Arthrinium* was recommended for use in unitary nomenclature (Réblová et al. 2016). However, due to several names with comparable sexual morphs to those of *Arthrinium* described as *A.montagnei*, the exact identity of *A.montagnei* remained uncertain (Hudson et al. 1976; Pintos et al. 2019; Pintos and Alvarado 2021). With the availability of sequence data of *A.montagnei*, Pintos and Alvarado (2022) revealed that *Apiospora* and *Arthrinium* are distinct genera. With most *Apiospora* species sharing similar morphologies, molecular phylogenetic information is necessary for accurate species identification (Pintos and Alvarado 2022).

The aim of the present study is to research new *Apiospora* samples found in western China, including one known species of *A.arundinis* and three new species and to describe them, based on morphological characters and phylogeny inferred from the combined ITS, LSU, *tef1* and *tub2* sequences dataset. To identify and compare these species with morphologically similar and phylogenetically related species, thorough analyses have been conducted.

## ﻿Materials and methods

### ﻿Sample collection and fungal isolation

Diseased leaves with dried dark brown spots of *Oenotherabiennis* and *Lophatherumgracile*, as well as diseased leaves with white round patches and black cracks of *Brunfelsiabrasiliensis* were collected from two locations in Yunnan Province: Lincang City (1547 m elevation; 23°52'12"N, 100°4'12"E) and Xishuangbanna City (763 m elevation; 22°1'48"N, 100°52'48"E). Dead plant culms of *Corylusyunnanensis* were collected in Ankang City (1683 m elevation; 33°26'37"N, 108°26'4"E), located in Shaanxi Province. All samples were placed in paper bags and transported to the laboratory for isolation. The samples were surface-sterilised by being exposed to 75% ethanol for one minute, followed by 1.25% sodium hypochlorite for three minutes, then another minute of exposure to 75% ethanol. The samples were then rinsed with distilled water for two minutes and dried on sterile filter paper. The affected portions of the leaves were excised into 0.5 × 0.5 cm fragments using a sterile razor blade. The fragments were then placed on to potato dextrose agar plates (PDA; containing 200 g potatoes, 20 g dextrose and 20 g agar per litre). The plates were incubated at a temperature of 25 °C to obtain pure cultures. All specimens were deposited at the Museum of Beijing Forestry University (**BJFC**) and all cultures were preserved at the China Forestry Culture Collection Center (**CFCC**).

### ﻿Morphological observation

The morphology of the isolates was examined by analysing sporulating axenic cultures cultivated on PDA in darkness at 25 °C. After a 7-day incubation period, colony diameters were measured and colony characters were recorded. Slide mounts were prepared in lactic acid or water, obtained from colonies sporulating on PDA. Observations were conducted using a Leica DM 2500 dissecting microscope (Wetzlar, Germany) and a Nikon Eclipse 80i compound microscope, equipped with differential interference contrast (DIC) illumination. Images were captured with a Nis DS-Ri2 camera and processed using the Nikon Nis Elements F4.30.01 software. For measurement purposes, 50 conidiogenous cells and conidia were randomly selected. Conidial length was measured from the base of the basal cell to the base of the apical appendage, while conidial width was measured at its widest point. Taxonomic novelties were deposited in MycoBank (http://www.mycobank.org).

### ﻿DNA extraction, PCR amplification and phylogenetic analyses

Genomic DNA was extracted from colonies grown on PDA using a cetyltrimethylammonium bromide (CTAB) method (Doyle and Doyle 1990). The extracted DNA products were stored at -20 °C until analysis. Four different loci were targeted for sequencing, including the nrDNA internal transcribed spacer regions 1 and 2 with the intervening 5.8S subunit (ITS), a partial sequence of the large subunit nrDNA subunit (LSU), a partial sequence of the translation elongation factor 1-alpha gene (*tef1*) and a partial sequence of the beta-tubulin gene (*tub2*). They were all amplified with the primer pairs and polymerase chain reaction (PCR) programme listed in Table 1.

**Table 1.** Gene regions and respective primer pairs used in the study.

Locus	PCR primers	PCR: thermal cycles: (Annealing temperature in bold)	Reference
ITS	ITS1/ITS4	(94 °C: 30 s, 55 °C: 30 s, 72 °C: 45 s) × 35 cycles	White et al. 1990
LSU	LR0R/LR5	(94 °C: 30 s, 48 °C: 50 s, 72 °C: 1 min 30 s) × 35 cycles	Cubeta et al. 1991
tef1	EF1-728F/EF2	(95 °C: 30 s, 51 °C: 30 s, 72 °C: 1 min) × 35 cycles	O’Donnell et al. 1998; Carbone and Kohn 1999
tub2	Bt-2a/Bt-2b	(95 °C: 30 s, 56 °C: 30 s, 72 °C: 1 min) × 35 cycles	Glass and Donaldson 1995

The PCR products were assayed by electrophoresis in 2% agarose gels. Amplified PCR products were sent to a commercial sequencing provider (Tsingke Biotechnology Co. Ltd., Beijing, China). The quality of the chromatograms was verified and nucleotide sequences were assembled using SeqMan v.7.1.0. Reference sequences from related publications (Wang et al. 2018; Pintos and Alvarado 2021; Samarakoon et al. 2022; Liu et al. 2023) were retrieved from the National Center for Biotechnology Information (NCBI; https://www.ncbi.nlm.nih.gov). Sequences were aligned on the web server using MAFFT at the web server (http://mafft.cbrc.jp/alignment/server) (Katoh et al. 2019) and further corrected manually utilising MEGA 7.0.21 (Kumar et al. 2016).

The phylogenetic analyses of the combined loci were performed using Maximum Likelihood (ML) and Bayesian Inference (BI) methods. To implement ML, RAxMLHPC BlackBox 8.2.10 (Stamatakis 2014) was used on the CIPRES Science Gateway portal (https://www.phylo.org) employing a GTR GAMMA substitution model with a total of 1000 bootstrap replicates. The Bayesian posterior probabilities (BPP) were determined by Markov Chain Monte Carlo (MCMC) sampling in MrBayes v.3.2.6 (Ronquist et al. 2012). Six simultaneous Markov chains were run for 1 million generations starting from random trees, sampling trees every 100^th^ generation. To ensure accuracy, 25% of aging samples were discarded, running until the average standard deviation of the split frequencies dropped below 0.01. The phylogram was visualised in FigTree v.1.3.1 (http://tree.bio.ed.ac.uk/software) and edited using Adobe Illustrator CS5 (Adobe Systems Inc., USA). The newly-generated nucleotide sequences were deposited in GenBank (Table 2).

**Table 2.** Isolates and GenBank accession numbers used in the phylogenetic analyses.

Species	Isolate/Strain	Host/ Substrate	Origin	GenBank accession numbers			
				ITS	LSU	*tef1*	*tub2*
*Apiosporaacutiapica*	KUMCC 20-0210 (Type)	*Bambusabambos*	China	MT946343	MT946339	MT947360	MT947366
*A.agari*	KUC 21333 (Type)	*Agarumcribrosum*	Korea	MH498520	MH498440	MH544663	MH498478
*A.aquatica*	MFLU 18-1628 (Type)	Submerged wood	China	MK828608	MK835806	NA	NA
*A.arctoscopi*	KUC 21331 (Type)	Egg of *Arctoscopusjaponicus*	Korea	MH498529	MH498449	MN868918	MH498487
*A.arundinis*	CBS 10612	Unkown substrate	Germany	KF144883	KF144927	KF145015	KF144973
	LX 1918	*Saccharumofficinarum*	China	MW534386	NA	MW584370	MZ090019
	**CFCC 58977**	***Brunfelsiabrasiliensis***	**China**	**OR125562**	**OR133584**	**OR139968**	**OR139976**
	**LS 107**	***Brunfelsiabrasiliensis***	**China**	**OR125563**	**OR133585**	**OR139969**	**OR139977**
*A.aurea*	CBS 24483 (Type)	Air	Spain	AB220251	KF144935	KF145023	KF144981
*A.balearica*	CBS 145129 (Type)	Poaceae	Spain	MK014869	MK014836	MK017946	MK017975
*A.bambusae*	ICPM 6889 (Type)	Bamboo	China	MK014874	MK014841	MK017951	MK017980
*A.bambusicola*	MFLUCC 20-0144 (Type)	*Schizostachyumbrachycladum*	Thailand	MW173030	MW173087	MW183262	
*A.biserialis*	CGMCC 320135 (Type)	Bamboo	China	MW481708	MW478885	MW522938	MW522955
*A.camelliae-sinensis*	LC 5007 (Type)	*Camelliasinensis*	China	KY494704	KY494780	KY705103	KY705173
*A.chromolaenae*	MFLUCC 17-1505 (Type)	*Chromolaenaodorata*	Thailand	MT214342	MT214436	MT235802	NA
*A.chiangraiense*	MFLUCC 21-0053 (Type)	Bamboo	Thailand	MZ542520	MZ542524	NA	MZ546409
*A.cordylinae*	GUCC 10027 (Type)	*Cordylinefruticosa*	China	MT040106	NA	MT040127	MT040148
***A.coryli***	**CFCC 58978 (Type)**	***Corylusyunnanensis***	**China**	**OR125564**	**OR133586**	**OR139974**	**OR139978**
	**CFCC 58979**	***Corylusyunnanensis***	**China**	**OR125565**	**OR133587**	**OR139975**	**OR139979**
*A.cyclobalanopsidis*	CGMCC 320136 (Type)	*Cyclobalanopsidisglauca*	China	MW481713	MW478892	MW522945	MW522962
*A.descalsii*	CBS 145130 (Type)	*Ampelodesmosmauritanicus*	Spain	MK014870	MK014837	MK017947	MK017976
*A.dichotomanthi*	LC 4950 (Type)	*Dichotomanthustristaniaecarpa*	China	KY494697	KY494773	KY705096	KY705167
*A.dongyingensis*	SAUCC 0302 (Type)	Bamboo	China	OP563375	OP572424	OP573264	OP573270
*A.esporlensis*	CBS 145136 (Type)	*Phyllostachysaurea*	Spain	MK014878	MK014845	MK017954	MK017983
*A.euphorbiae*	IMI 285638b	*Bambusa*	Bangladesh	AB220241	AB220335	NA	AB220288
*A.fermenti*	KUC21289 (Type)	Seaweed	Korea	MF615226	MF615213	MH544667	MF615231
*A.gaoyouense*	CFCC 52301 (Type)	*Phragmitesaustralis*	China	MH197124	NA	MH236793	MH236789
*A.garethjonesii*	JHB004 (Type)	Bamboo	China	KY356086	KY356091	NA	NA
*A.gelatinosa*	HKAS 111962 (Type)	Bamboo	China	MW481706	MW478888	MW522941	MW522958
*A.guiyangensis*	HKAS 102403 (Type)	Poaceae	China	MW240647	MW240577	MW759535	MW775604
*A.guizhouensis*	LC 5322 (Type)	Air in karst cave	China	KY494709	KY494785	KY705108	KY705178
*A.hainanensis*	SAUCC 1681 (Type)	Bamboo	China	OP563373	OP572422	OP573262	OP573268
*A.hispanicum*	IMI 326877 (Type)	Maritime sand	Spain	AB220242	AB220336	NA	AB220289
*A.hydei*	CBS 114990 (Type)	*Bambusatuldoides*	China	KF144890	KF144936	KF145024	KF144982
*A.hyphopodii*	MFLUCC 15-0003 (Type)	Bamboo	China	KR069110	NA	NA	NA
*A.ibericum*	AP 10118 (Type)	*Arundodonax*	Portugal	MK014879	MK014846	MK017955	MK017984
*A.intestini*	CBS 135835 (Type)	Gut of grasshopper	India	KR011352	MH877577	KR011351	KR011350
*A.italicum*	CBS 145138 (Type)	*Arundodonax*	Italy	MK014880	MK014847	MK017956	MK017985
*A.jatrophae*	CBS 134262 (Type)	*Jatrophapodagrica*	India	JQ246355	NA	NA	NA
*A.jiangxiensis*	LC 4577 (Type)	*Maesa* sp.	China	KY494693	KY494769	KY705092	KY705163
*A.kogelbergensis*	CBS 113333 (Type)	Restionaceae	South Africa	KF144892	KF144938	KF145026	KF144984
*A.koreanum*	KUC 21332 (Type)	Egg of *Arctoscopusjaponicus*	Korea	MH498524	MH498444	MH544664	MH498482
*A.lageniformis*	KUC 21686 (Type)	*Phyllostachysnigra*	Korea	ON764020	ON787759	ON806624	ON806634
*A.locuta-pollinis*	LC 11683 (Type)	*Brassicacampestris*	China	MF939595	NA	MF939616	MF939622
*A.longistroma*	MFLUCC 11-0481 (Type)	Bamboo	Thailand	KU940141	KU863129	NA	NA
***A.lophatheri***	**CFCC 58975 (Type)**	***Lophatherumgracile***	**China**	**OR125566**	**OR133588**	**OR139970**	**OR139980**
	**CFCC 58976**	***Lophatherumgracile***	**China**	**OR125567**	**OR133589**	**OR139971**	**OR139981**
*A.malaysiana*	CBS 102053 (Type)	*Macarangahullettii* stem colonised by ants	Malaysia	KF144896	KF144942	KF145030	KF144988
*A.marianiae*	AP18219 (Type)	*Phleumpratense*	Spain	ON692406	ON692422	ON677180	ON677186
*A.marii*	CBS 49790 (Type)	Atmosphere, pharmaceutical excipients, home dust and beach sands	Spain	MH873913	KF144947	KF145035	KF144993
*A.marinum*	KU 21328 (Type)	Seaweed	China	MH498538	MH498458	MH544669	MH498496
*A.mediterranea*	IMI 326875 (Type)	Air	Spain	AB220243	AB220337	NA	AB220290
*A.minutisporum*	17E-042 (Type)	Soil	Korea	LC517882	NA	LC518889	LC518888
*A.montagnei*	AP 301120 (Type)	*Arundomicrantha*	Spain	ON692408	ON692424	ON677182	ON67718
*A.mori*	MFLU 18-2514 (Type)	*Morusaustralis*	China	MW114313	MW114393	NA	NA
*A.mukdahanensis*	MFLUCC 22-0056 (Type)	Bambusoideae	Thailand	OP377735	OP377742	OP381089	NA
*A.multiloculata*	MFLUCC 21-0023 (Type)	*Bambusae*	Thailand	OL873137	OL873138	NA	OL874718
*A.mytilomorpha*	DAOM 214595 (Type)	*Andropogon*	India	KY494685	NA	NA	NA
*A.neobambusae*	LC 7106 (Type)	Bamboo	China	KY494718	KY494794	KY806204	KY705186
*A.neochinensis*	CFCC 53036 (Type)	*Fargesiaqinlingensis*	China	MK819291	NA	MK818545	MK818547
*A.neogarethjonesii*	HKAS 102408 (Type)	*Bambusae*	China	MK070897	MK070898	NA	NA
*A.neosubglobosa*	JHB007 (Type)	Bamboo	China	KY356090	KY356095	NA	NA
*A.obovatum*	LC4940 (Type)	*Lithocarpus*	China	KY494696	KY494772	KY705095	KY705166
***A.oenotherae***	**CFCC 58972 (Type)**	***Oenotherabiennis***	**China**	**OR125568**	**OR133590**	**OR139972**	**OR139982**
	**LS 395**	***Oenotherabiennis***	**China**	**OR125569**	**OR133591**	**OR139973**	**OR139983**
*A.ovata*	CBS 115042 (Type)	*Arundinariahindsii*	China	KF144903	KF144950	KF145037	KF144995
*A.paraphaeosperma*	MFLUCC13-0644 (Type)	*Bambusa*	Thailand	KX822128	KX822124	NA	NA
*A.phragmitis*	CBS 135458 (Type)	*Phragmitesaustralis*	Italy	KF144909	KF144956	KF145043	KF145001
*A.phyllostachydis*	MFLUCC 18-1101 (Type)	*Phyllostachysheteroclada*	China	MK351842	MH368077	MK340918	MK291949
*A.piptatheri*	CBS 145149 (Type)	*Piptatherummiliaceum*	Spain	MK014893	MK014860	MK017969	NA
*A.pseudomarii*	GUCC 10228 (Type)	*Aristolochiadebilis*	China	MT040124	NA	MT040145	MT040166
*A.pseudohyphopodii*	KUC 21680 (Type)	*Phyllostachyspubescens*	Korea	ON764026	ON787765	ON806630	ON806640
*A.pseudoparenchymaticum*	LC 7234 (Type)	Bamboo	China	KY494743	KY494819	KY705139	KY705211
*A.pseudorasikravindrae*	KUMCC 20-0208 (Type)	*Bambusadolichoclada*	China	MT946344	NA	MT947361	MT947367
*A.pseudosinensis*	CBS 135459 (Type)	Bamboo	Netherlands	KF144910	KF144957	KF145044	NA
*A.pseudospegazzinii*	CBS 102052 (Type)	*Macarangahullettii*	Malaysia	KF144911	KF144958	KF145045	KF145002
*A.pterosperma*	CPC 20193 (Type)	*Lepidospermagladiatum*	Australia	KF144913	KF144960	KF145046	KF145004
*A.pusillisperma*	KUC 21321 (Type)	Seaweed	Korea	MH498533	MH498453	MN868930	MH498491
*A.qinlingense*	CFCC 52303 (Type)	*Fargesiaqinlingensis*	China	MH197120	NA	MH236795	MH236791
*A.rasikravindrae*	NFCCI 2144 (Type)	Soil in karst cave	China	JF326454	NA	NA	NA
*A.sacchari*	CBS 21230	*Phragmitesaustralis*	Korea	KF144919	KF144965	KF145050	KF145008
*A.saccharicola*	CBS 19173	Air	Netherlands	KF144920	KF144966	KF145051	KF145009
*A.sargassi*	KUC21228 (Type)	*Sargassumfulvellum*	Korea	KT207746	KT207696	MH544677	KT207644
*A.sasae*	CBS 146808 (Type)	*Sasaveitchii*	Netherlands	MW883402	MW883797	MW890104	MW890120
*A.septata*	CGMCC 320134 (Type)	Bamboo	China	MW481711	MW478890	MW522943	MW522960
*A.serenensis*	IMI 326869 (Type)	Food, pharmaceutical excipients, atmosphere and home dust	Spain	AB220250	AB220344	NA	AB220297
*A.setariae*	CFCC 54041 (Type)	*Setariaviridis*	China	MT492004	NA	NA	NA
*A.setostroma*	KUMCC 19-0217 (Type)	Bambusoideae	China	MN528012	MN528011	MN527357	NA
*A.sichuanensis*	HKAS 107008 (Type)	Poaceae	China	MW240648	MW240578	MW759536	MW775605
*A.sorghi*	URM 93000 (Type)	*Sorghumbicolor*	Brazil	MK371706	NA	NA	MK348526
*A.sphaerosperma*	CBS114314 (Type)	*Hordeumvulgare*	Iran	KF144904	KF144951	KF145038	KF144996
*A.stipae*	CBS 146804 (Type)	*Stipagigantea*	Spain	MW883403	MW883798	MW890082	MW890121
*A.subglobosa*	MFLUCC 11-0397 (Type)	Bamboo	Thailand	KR069112	KR069113	NA	NA
*A.subrosea*	LC7292 (Type)	Bamboo	China	KY494752	KY494828	KY705148	KY705220
*A.taeanensis*	KUC21322 (Type)	Seaweed	Korea	MH498515	MH498435	MH544662	MH498473
*A.thailandica*	MFLUCC 15-0202 (Type)	Rotten wood	China	KU940145	KU863133	NA	NA
*A.vietnamense*	IMI 99670 (Type)	*Citrussinensis*	Vietnam	KX986096	KX986111	NA	KY019466
*A.xenocordella*	CBS 47886 (Type)	Soil from roadway	Zimbabwe	KF144925	KF144970	KF145055	KF145013
*A.yunnana*	MFLUCC 15-0002 (Type)	Bamboo	China	KU940147	KU863135	NA	NA
*Arthriniumcrenatum*	CBS 146353B (Type)	Grass	France	MW208931	MW208861	MW221917	MW221923

Notes: Strains in this study are marked in bold. NA = not available.

## ﻿Results

### ﻿Phylogeny

The combined ITS, LSU, *tef1* and *tub2* dataset comprised 99 strains, including eight newly-sequenced strains, with *Arthriniumcrenatum* (CBS 146353) as the outgroup taxon. Multi-locus sequences contain 2,709 characters including gaps with ITS (1–610), LSU (611–1399), *tef1* (1400–1948) and *tub2* (1949–2691). Of these characters, 1,635 were constant, 367 were variable and parsimony-uninformative and 707 were parsimony-informative. For ML analysis, the matrix had 1,192 distinct alignment patterns. Estimated base frequencies were A = 0.229212, C = 0.248907, G = 0.263837, T = 0.258044; substitution rates: AC = 1.129211, AG = 2.936388, AT = 0.925501, CG = 0.917970, CT = 4.199729, GT = 1.0; gamma distribution shape parameter: α = 0.250690; and likelihood value of ln: -22 496.696950.

**Figure 1.**
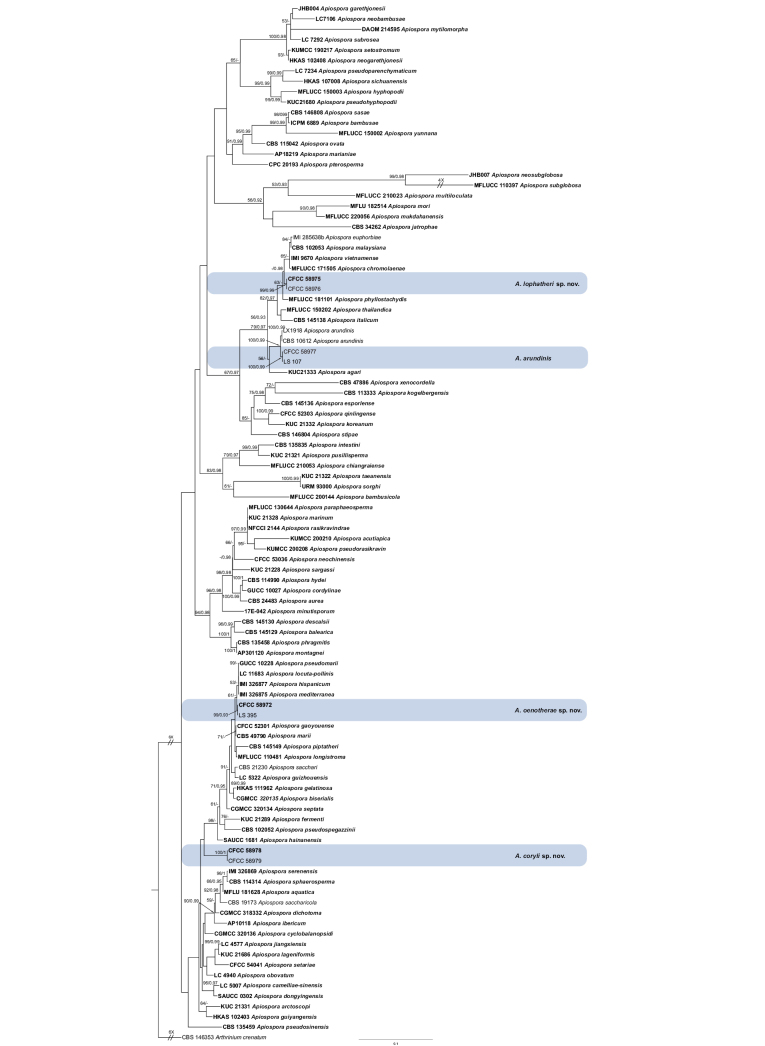
Phylogram of *Apiospora*, based on combined ITS, LSU, *tef1* and *tub2* genes. ML bootstrap support values (≥ 50%) and Bayesian posterior probability (≥ 0.90) are shown as first and second position above nodes, respectively. Strains from this study are shown in blue boxes, ex-type or ex-epitype cultures are indicated in bold face. Some branches were shortened according to the indicated mulipliers.

The ML tree topology agreed with the BI analysis and, therefore, only the ML tree is presented (Fig. 1). The strains obtained in this study were categorised into four clades, representing one known species and three new species (Fig. 1). The known species is *A.arundinis* and three new species are now recognised as *A.coryli*, *A.lophatheri* and *A.oenotherae*.

### ﻿Taxonomy

#### ﻿ Apiospora arundinis

Taxon classificationFungiXylarialesApiosporaceae

(Corda) Pintos & P. Alvarado, Fungal Systematics and Evolution 7: 205 (2021)

05626E62-5E3C-55EF-9D8A-04B58A926EA0

Fig. 2

##### Description.

***Asexual morph***: Mycelium consisting of smooth, hyaline, branched, septate, 1.1–5.9 µm diam. hyphae (n = 20). Conidiophores reduced to conidiogenous cells. Conidiogenous cells subglobose to ampulliform, erect, blastic, aggregated in clusters on hyphae, smooth, branched, 3.4–9.4 × 1.5–6.4 µm, mean (± SD): 6.8 (± 1.6) × 3.9 (± 1.3) µm (n = 50). Conidia globose, subglobose to lenticular, with a longitudinal germ slit, occasionally elongated to ellipsoidal, brown to dark brown, smooth to finely roughened, 6.4–10.4 × 5.2–8.3 µm, mean (± SD): 7.7 (± 0.6) × 6.8 (± 0.7) µm, L/W = 1.0–1.5 (n = 50). ***Sexual morph***: Undetermined.

**Figure 2.**
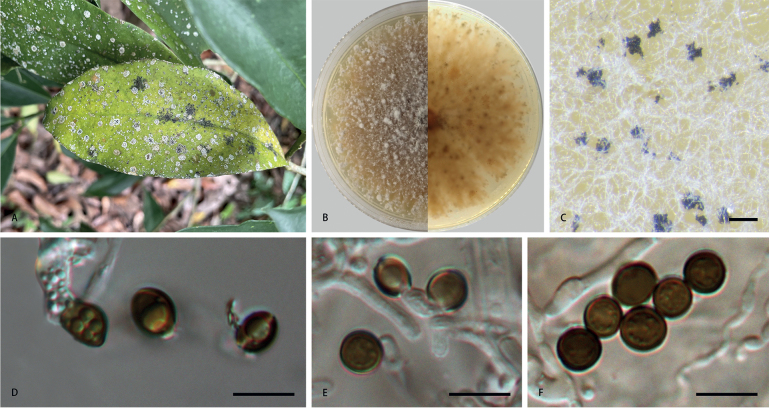
*Apiosporaarundinis* (**CFCC 58977**) **A** leaf of host plant **B** colony on PDA **C** conidiomata formed in culture **D, E** conidiogenous cells giving rise to conidia **F** conidia. Scale bars: 1000 µm (**C**); 10 µm (**D–F**).

##### Culture characteristics.

On PDA, colonies thick and dense, margin undulate and irregular, pale yellow pigment diffused into medium, surface with patches of iron-grey aerial mycelia, reverse yellowish-brown, mycelia white to grey, sporulation on hyphae, reaching 9 cm in 7 days at 25 °C.

##### Specimens examined.

China, Yunnan Province: Xishuangbanna Botanical Garden, on diseased leaves of *Brunfelsiabrasiliensis*, 6 June 2022, S.J. Li, BJFC-S1918; living cultures CFCC 58977, LS 107).

##### Notes.

In this study, two isolates clustered together with the culture of *A.arundinis* with high-support values (ML/BI = 100/0.99)in the multi-locus phylogenetic tree (Fig. 1). Thus, these isolates were identified as *A.arundinis* and *Brunfelsiabrasiliensis* as a new host record for this species. *Apiosporaarundinis* was introduced from *Phyllostachyspraecox*, *Castaneamollissima* and *Saccharumofficinarum* in China (Chen et al. 2014; Jiang et al. 2021; Liao et al. 2022). Comparing with the description from Chen et al. (2014) (5–7 × 2–4 µm), Jiang et al. (2021) (3–4 µm) and Liao et al. (2022) (4.5–7.4 × 3.3–4.4 µm), the conidia in this study show larger sizes (6.4–10.4 × 5.2–8.3 µm). These differences may result from different host and habitat.

#### ﻿ Apiospora coryli

Taxon classificationFungiXylarialesApiosporaceae

S.J. Li & C.M. Tian sp. nov.

C96D4D20-1AAE-5841-8C28-0671DF4611BB

849126

Fig. 3

##### Type.

China, Shanxi Province: Ankang City, Huoditang Forest Farm, on dead plant culms of *Corylusyunnanensis*, 16 July 2021, R. Yuan & S.J. Li, holotype BJFC-S1920, ex-type living cultures CFCC 58978, CFCC 58979.

##### Etymology.

Named after the host from which it was isolated.

##### Description.

***Asexual morph***: Derived from sporulated cultures on PDA, hyphae hyaline, branched, septate, 1.1–5.2 µm diam. Conidiophores reduced to conidiogenous cells. Conidiogenous cells erect, aggregated in clusters on hyphae, hyaline to pale brown, smooth, doliiform to clavate or lageniform, 2.6–10.6 × 2.1–5.8 µm, mean (± SD): 5.5 (± 2.4) × 3.4 (± 1.1) µm (n = 50). Conidia brown to dark brown, globose to subglobose, oval or irregular, smooth to finely roughened, guttulate, usually with a longitudinal germ slit, 7.4–18.4 × 6.2–12.5 µm, mean (± SD): 10.8 (± 1.7) × 9.4 (± 1.3) µm, L/W = 0.8–1.6 (n = 50). ***Sexual morph***: Undetermined.

**Figure 3.**
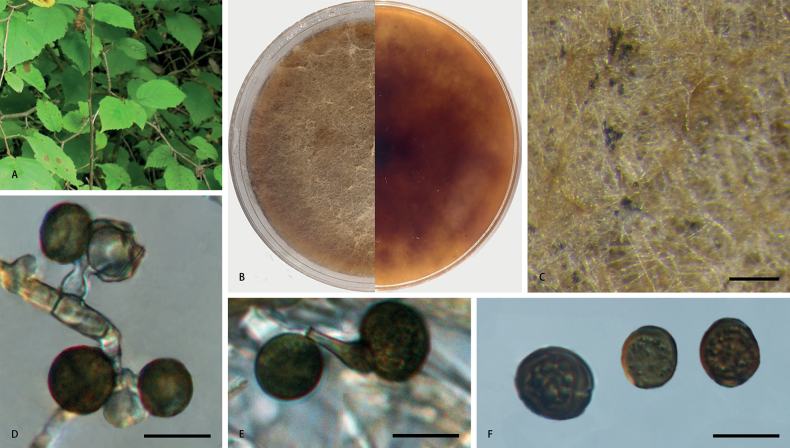
*Apiosporacoryli* (**CFCC 58978, ex-holotype culture**) **A** leaf of host plant **B** colony on PDA **C** conidiomata formed in culture **D, E** conidiogenous cells giving rise to conidia **F** conidia. Scale bars: 1000 µm (**C**); 10 µm (**D–F**).

##### Culture characteristics.

On PDA, colonies circular, flat, entire margin, thick and cottony, concentrically spreading with aerial mycelium, margin regular, reddish-brown pigment diffused into medium, surface dark yellowish-brown, reverse dark reddish-brown to yellowish-brown from the centre, mycelia white to pale umber, sporulation on hyphae, reaching 9 cm in 7 days at 25 °C.

##### Notes.

Strains of *A.coryli* constitutes a distinct clade, but there is poor support value in concatenated gene trees (Fig. 1). The most prominent distinguishing characteristic is the production of reddish-brown pigments on the culture medium.

#### ﻿ Apiospora lophatheri

Taxon classificationFungiXylarialesApiosporaceae

S.J. Li & C.M. Tian sp. nov.

2CD5427F-4DD4-56BE-BEC8-CEC89EE63775

849123

Fig. 4

##### Type.

China, Yunnan Province, Xishuangbanna Primeval Forest Park, on diseased leaves of *Lophatherumgracile*, 4 June 2022, S.J. Li, holotype BJFC-S1917; ex-type living cultures CFCC 58975, CFCC 58976.

##### Etymology.

Named after the host from which it was isolated.

##### Description.

***Asexual morph***: Sporulated on PDA, mycelium consisting of hyaline, smooth, branched, septate hyphae 1.0–5.2 µm in diam. (n = 20). Conidiophores reduced to conidiogenous cells. Conidiogenous cells aggregated in clusters on hyphae, hyaline to pale brown, smooth, doliiform, clavate to ampulliform, 2.2–11.9 × 2.2–4.9 µm, mean (± SD): 6.4 (± 2.5) × 3.4 (± 0.6) µm (n = 50). Conidia globose, subglobose to lenticular, with a longitudinal germ slit, olive to dark brown, smooth to finely roughened and two or more conidia are produced on each conidiogenous cell, 5.1–8.9 × 4.6–7.7 µm, mean (± SD): 6.5 (± 0.8) × 5.9 (± 0.7) µm, L/W = 1.0–1.4 (n = 50). ***Sexual morph***: Undetermined.

**Figure 4.**
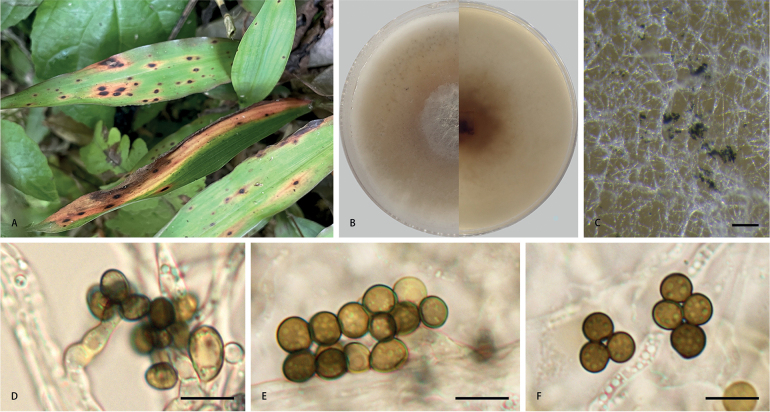
*Apiosporalophatheri* (**CFCC 58975, ex-holotype culture**) **A** leaf of host plant **B** colony on PDA **C** conidiomata formed in culture **D** conidiogenous cells giving rise to conidia **E, F** conidia. Scale bars: 1000 µm (**C**); 10 µm (**D–F**).

##### Culture characteristics.

On PDA, colonies flat, spreading, margin circular, thick, concentrically spreading with aerial mycelium, surface light greyish-brown, reverse tawny pigment diffused in media, mycelia white to grey and pale brown, sporulation on hyphae, reaching 9 cm in 7 days at 25 °C.

##### Notes.

Phylogenetic analysis indicated that *Apiosporalophatheri* is closely related to a clade comprising *A.chromolaenae*, *A.euphorbiae*, *A.italicum*, *A.malaysiana*, *A.phyllostachydis*, *A.thailandica* and *A.vietnamense* (Fig. 1). We compared the new species with phylogenetically similar taxa, based on morphological differences (Table 3) and base pair differences (Table 4). *A.lophatheri* can be differentiated from *A.chromolaenae* by its wider conidiogenous cells (2.2–11.9 × 2.2–4.9 µm vs. 6.5–12 × 1–2 µm) (from *Euphorbia* sp.; collected in Zambia; Ellis (1965)) and by 18 gene base pair differences (17/529 in ITS, 1/838 in LSU). *A.lophatheri* differs from *A.euphorbiae* by its larger olive to dark brown conidia (5.1–8.9 × 4.6–7.7 µm vs. 4–5.5 × 3–4 µm) (from *Euphorbia* sp.; collected in Zambia; Ellis (1965)), with nucleotide differences in ITS as 3/529, in LSU as 2/318, in *tub2* as 22/801. *A.italicum* has smaller conidia (4–6 × 3–4 µm) (from *Arundodonax*; collected in Italy; Pintos et al. (2019)) and has 125 nucleotides differences (41/552 in ITS, 2/828 in LSU, 27/432 in *tef1*, 55/838 in *tub2*). Additionally, *A.lophatheri* is distinguished from *A.malaysiana* by having larger globose or subglobose conidia (5.1–8.9 × 4.6–7.7 µm vs. 5–6 × 3–4 µm) (from *Macarangahullettii*; collected in Malaysia; Crous and Groenewald (2013)), with 43 nucleotide differences (3/529 in ITS, 1/838 in LSU, 18/424 in *tef1*, 21/801 in *tub2*). *A.lophatheri* differs from *A.phyllostachydis* by its relatively shorter conidiogenous cells (2.2–11.9 × 2.2–4.9 µm vs. 20–55 × 1.5–2.5 µm) (from *Phyllostachysheteroclada*; collected in China; Yang et al. (2019)) and by 48 nucleotides differences (7/529 in ITS, 3/838 in LSU, 12/424 in *tef1*, 26/795 in *tub2*). *A.lophatheri* can be differentiated from *A.thailandica* by having shorter conidiogenous cells (2.2–11.9 × 2.2–4.9 µm vs. 11.5–39 × 2–3.5 µm) (from bamboo; collected in Thailand; Dai et al. (2017)) and by 12 nucleotides differences (9/529 in ITS, 3/828 in LSU). The conidia of *A.lophatheri* are significantly wider and paler-coloured than those of *A.vietnamense* (5.1–8.9 × 4.6–7.7 µm vs. 5–6 × 3–4 µm) (from *Citrussinensis*; collected in Vietnam; Wang et al. (2018)) and there are 7 nucleotides differences between the two species (2/526 in ITS, 2/803 in LSU, 3/315 in *tub2*). Therefore, *A.lophatheri* is described as a new species, based on phylogeny and morphological comparison.

**Table 3.** Summary of morphology of new *Apiospora* species and phylogenetic related species.

Species	Isolation source	Country	Conidiogenous cells (µm)	Conidia in surface view		Conidia in side view		References
				Shape	Diam (μm)	Shape	Diam (μm)	
*A.gaoyouense*	*Phragmitesaustralis*	China	1–2 × 2–3	globose to elongate ellipsoid	5–8	lenticular	4–8	Jiang et al. (2018)
*A.hispanicum*	Maritime sand	Spain	–	globose to ellipsoid	7.5–8.5 × 6–7.5	lenticular	6.5	Larrondo (1992)
*A.locuta-pollinis*	*Brassicacampestris*	China	3–7.5 × 3–6	globose to elongate ellipsoid	8–15× 5–9.5	–	–	Zhao et al. (2018)
*A.longistroma*	Bamboo	Thailand	–	asexual morph: Undetermined	–	–	–	Dai et al. (2017)
*A.marii*	Beach sand/ Poaceae	Spain	5–10 × 3–4.5	globose to elongate ellipsoid	8–10(−13)	lenticular	(5–)6(−8)	Crous and Groenewald (2013)
*A.mediterranei*	Airborn spore/ grass	Spain	–	lentiform	9–9.5 × 7.5–9	–	–	Larrondo (1992)
*A.oenotherae*	*Oenotherabiennis*	China	2.0–14.2 × 1.1–4.9	globose, subglobose to lenticular	6.6–13.9 × 5.5–10.1	–	–	This study
*A.piptatheri*	*Piptatherummiliaceum*	Spain	6–27 × 2–5	globose to elongate ellips oid	6–8 × 3–5	lenticular	4.5–6	Pintos et al. (2019)
*A.pseudomarii*	*Aristolochiadebilis*	China	8–13 × 2.5–5	subglobose to ellipsoid	6–9 × 4.5–6	–	–	Chen et al. (2021)
*A.chromolaenae*	*Chromolaenaodorata*	Thailand	6.5–12 × 1–2	elongated, broadly fliform to ampulliform	4–6×4.5–6.5	–	–	Mapook et al. (2020)
*A.euphorbiae*	*Bambusa*	Bangladesh	–	circular or nearly circular	(4–)4.7(–5.5)	lenticular	(3–)3.2(–4)	Sharma et al. (2014)
*A.italicum*	*Arundodonax*	Italy	(3–)4–7(–9) × (1.5–)2–3(–5)	globose	4–6×3–4	lenticular	–	Pintos et al. (2019)
*A.lophatheri*	*Lophatherumgracile*	China	2.2–11.9 × 2.2–4.9	globose, subglobose to lenticular	5.1–8.9 × 4.6–7.7	–	–	This study
*A.malaysiana*	*Macarangahullettii*	Malaysia	4–7 × 3–5	globose	5–6	lenticular	3–4	Crous and Groenewald (2013)
*A.phyllostachydis*	*Phyllostachysheteroclada*	China	20–55 × 1.5–2.5	globose to subglobose, oval or irregular	5–6 × 4–6	–	–	Yang et al. (2019)
*A.thailandicum*	Bamboo	Thailand	11.5–39 × 2–3.5	globose to subglobose, elongated to ellipsoidal	5–9 × 5–8	–	–	Dai et al. (2017)
*A.vietnamense*	*Citrussinensis*	Vietnam	4–7 × 3–5	globose	5–6	lenticular	3–4	Wang et al. (2017)

**Table 4.** DNA base differences comparing *Apiosporalophatheri* sequences and sequences from related species.

Taxa	Loci	Nucleotides difference without gaps	Rates of base pair differences
*A.chromolaenae*	ITS	17/529 (40, 102, 108, 109, 110, 111, 112, 113, 114, 115, 116, 117, 118, 119, 120, 121, 122)	3.21%
	LSU	1/838 (426)	0.12%
*A.euphorbiae*	ITS	3/515 (26, 88, 89)	0.58%
	LSU	2/318 (146, 306)	0.63%
	*tub2*	22/801 (95, 96, 123, 151, 154, 163, 166, 182, 185, 193, 216, 237, 312, 347, 372, 429, 453, 454, 474, 559, 569, 574)	2.75%
*A.italicum*	ITS	41/552 (40, 82, 93, 94, 95, 96, 97, 98, 99, 100, 101, 102, 103, 104, 105, 106, 107, 108, 109, 110, 111, 112, 113, 114, 115, 116, 117, 118, 119, 120, 121, 122, 132, 165, 177, 180, 205, 207, 213, 487, 529)	7.43%
	LSU	2/828 (406, 416)	0.24%
	*tef1*	27/432 (16, 18, 19, 20, 21, 22, 23, 24, 25, 27, 35, 46, 53, 60, 75, 80, 90, 102, 119, 123, 125, 172, 210, 211, 240, 248, 272)	6.25%
	*tub2*	55/838 (5, 29, 44, 45, 46, 92, 99, 119, 121, 122, 126, 155, 157, 171, 185, 188, 193, 194, 196, 198, 202, 297, 219, 229, 240, 265, 315, 338, 358, 363, 367, 368, 382, 384, 386, 390, 403, 407, 412, 430, 434, 454, 463, 465, 467, 480, 491, 499, 502, 556, 564, 580, 642, 756, 757)	6.56%
*A.malaysiana*	ITS	3/529 (40, 102, 103)	0.57%
	LSU	1/838 (426)	0.12%
	*tef1*	18/424 (15, 16, 19, 27, 29, 38, 52, 56, 82, 83, 91, 93, 95, 111, 115, 202, 203, 264)	4.25%
	*tub2*	21/801 (95, 96, 123, 151, 154, 163, 166, 182, 185, 193, 216, 237, 312, 347, 372, 429, 453, 474, 559, 569, 574)	2.62%
*A.phyllostachydis*	ITS	7/529 (40, 44, 85, 102, 106, 433, 500)	1.32%
	LSU	3/838 (7,8,9)	0.36%
	*tef1*	12/424 (16, 19, 26, 27, 51, 52, 53, 111, 197, 202, 203, 264)	2.83%
	*tub2*	26/795 (35, 52, 55, 84, 89, 112, 116, 147, 151, 175, 178, 186, 209, 211, 231, 329, 352, 354, 360, 462, 469, 489, 570, 572, 575, 608)	3.27%
*A.thailandicum*	ITS	9/529 (40, 82, 102, 107, 122, 175, 177, 183, 501)	1.70%
	LSU	3/828 (5, 416, 434)	0.36%
*A.vietnamense*	ITS	2/526 (37, 99)	0.38%
	LSU	2/803 (237, 391)	0.25%
	*tub2*	3/315 (72, 82, 87)	0.95%

#### ﻿ Apiospora oenotherae

Taxon classificationFungiXylarialesApiosporaceae

S.J. Li & C.M. Tian sp. nov.

4E48C012-D878-50A9-B0E6-70877ECBD3A5

849125

Fig. 5

##### Type.

China, Yunnan Province, Lincang City Triangle Plum Garden, on diseased leaves of *Oenotherabiennis*, 26 April 2022, S.J. Li, holotype BJFC-S1919, ex-type living cultures CFCC 58972, LS 395.

##### Etymology.

Named after the host from which it was isolated.

##### Description.

***Asexual morph***: Hyphae hyaline, branched, septate, 1.2–4.8 µm in diam. (n = 20). Conidiophores reduced to conidiogenous cells. Conidiogenous cells smooth, ampulliform to doliiform, 2.0–14.2 × 1.1–4.9 µm, mean (± SD): 5.4 (± 2.9) × 3.1 (± 1.1) µm (n = 50). Conidia globose, subglobose to lenticular, with a longitudinal germ slit, occasionally elongated to ellipsoidal, colourless to dark brown, smooth to finely roughened, 6.6–13.9 × 5.5–10.1 µm, mean (± SD): 8.9 (± 1.2) × 7.8 (± 1.1) µm, L/W = 1.0–1.5 (n = 50). ***Sexual morph***: Undetermined.

**Figure 5.**
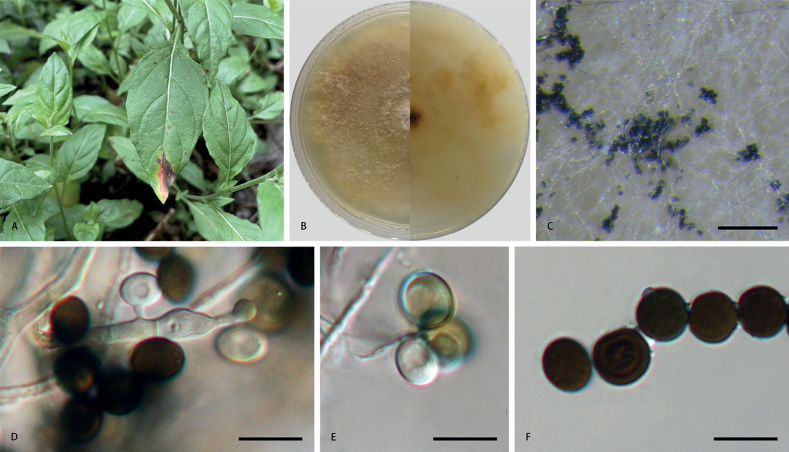
*Apiosporaoenotherae* (**CFCC 58972, ex-holotype culture**) **A** leaf of host plant **B** colony on PDA **C** conidiomata formed in culture **D, E** conidiogenous cells giving rise to conidia **F** conidia. Scale bars: 1000 µm (**C**); 10 µm (**D–F**).

##### Culture characteristics.

On PDA, colonies thick, concentrically spreading with aerial mycelium, circular, margin irregular, yellow to pale green pigment diffused into medium, surface with aerial mycelia, the reverse lightly pigmented with a few dark yellow patches, mycelia white to grey, sporulation occurs after 10 days, reaching 9 cm in 7 days at 25 °C.

##### Notes.

*Apiosporaoenotherae* belongs to the large clade, where it shows a relationship with *A.gaoyouense*, *A.hispanicum*, *A.locuta-pollinis*, *A.longistroma*, *A.marii*, *A.mediterranei*, *A.piptatheri* and *A.pseudomarii* (Fig. 1), but differs in distinct morphological characters (Table 3) and nucleotide differences (Table 5). *A.oenotherae* differs from *A.gaoyouense* by its production of significantly conidiogenous cells (2.0–14.2 × 1.1–4.9 µm vs. 1–2 × 2–3 μm) (from *Phragmitesaustralis*; collected in China; Jiang et al. (2018)) and the presence of 30 distinct nucleotide positions (9/583 in ITS, 12/413 in *tef1*, 9/784 in *tub2*). *A.oenotherae* is distinct from *A.hispanicum* in producing larger conidial cells (6.6–13.9 × 5.5–10.1 µm vs. 7.5–8.5 × 6.2–7.6 µm) (from maritime sand; collected in Spain; Larrondo and Calvo (1992)) and in 30 nucleotides differences (1/539 in ITS, 1/320 in LSU, 28/796 in *tub2*). *A.oenotherae* differs from *A.locuta-pollinis* by its production of significantly conidiogenous cells (2.0–14.2 × 1.1–4.9 µm vs. 3–7.5 × 3–6 μm) (from hive-stored pollen; collected in China; Zhao et al. (2018)) and by the presence of 19 distinct nucleotide positions (1/539 in ITS, 7/416 in *tef1*, 11/485 in *tub2*). *A.longistroma* can be distinguished by growth rate, growing slowly on PDA, reaching 60 mm in 4 weeks (from bamboo; collected in Thailand; Dai et al. (2017)) and by the presence of 8 distinct nucleotide positions (6/572 in ITS, 2/840 in LSU). Moreover, *A.mari* produces elongated cells intermingled amongst conidia (from beach sand; collected in Spain; Crous and Groenewald (2013)), but *A.oenotherae* does not and can be distinguished by the presence of 23 distinct nucleotide positions (1/539 in ITS, 10/414 in *tef1*, 12/787 in *tub2*). Strains of *A.mediterranei* were isolated from pharmaceutical excipient, air-borne and on grass in Spain, while those of *A.oenotherae* collected from *Oenotherabiennis* in China. There are no discernible morphological characters distinguishing these species, but the elongated stem branches and the presence of 30 distinct nucleotide positions (1/539 in ITS, 1/320 in LSU, 28/796 in *tub2*) serve as clear indicators of their distinct and phylogenetically well-separated taxa. *A.oenotherae* differs from *A.piptatheri* because of its wider conidial cells (6.6–13.9 × 5.5–10.1 µm vs. 6–8 × 3–5 μm) (from *Piptatherummiliaceum*; collected in Spain; Pintos et al. (2019)) and the presence of 14 distinct nucleotide positions (10/528 in ITS, 4/827 in LSU). It also differentiates from *A.pseudomarii* through the production of notably wider conidial cells (6.6–13.9 × 5.5–10.1 µm vs. 6–9 × 4.5–6 µm) and through 12 unique nucleotide positions (5/556 in *tef1*, 7/416 in *tub2*) (from *Aristolochiadebilis*; collected in China; Chen et al. (2021)).

**Table 5.** DNA base differences comparing *Apiosporaoenotherae* sequences and sequences from related species.

Taxa	Loci	Nucleotides difference without gaps	Rates of base pair differences
*A.gaoyouense*	ITS	9/583 (9, 10, 22, 36, 533, 535, 544, 555, 557)	1.54%
	*tef1*	12/413 (34, 48, 56, 57, 69, 90, 122, 129, 134, 170, 226, 228)	2.91%
	*tub2*	9/784 (538, 760, 766, 767, 768, 771, 775, 781, 782)	1.15%
*A.hispanicum*	ITS	1/539 (528)	0.19%
	LSU	1/320 (13)	0.31%
	*tub2*	28/796 (30, 186, 539, 761, 766, 767, 768, 769, 770, 771, 772, 773, 774, 775, 776, 777, 778, 779, 780, 781, 782, 783, 784, 785, 786, 787, 792, 794)	3.52%
*A.locuta-pollinis*	ITS	1/539 (528)	0.19%
	*tef1*	7/416 (33, 38, 94, 173, 177, 212, 258)	1.68%
	*tub2*	11/485 (237, 459, 465, 466, 467, 470, 474, 480, 481, 483, 485)	2.27%
*A.longistroma*	ITS	6/572 (20, 30, 38, 177, 213, 530)	1.05%
	LSU	2/840 (655, 825)	0.24%
*A.marii*	ITS	1/539 (528)	0.19%
	*tef1*	10/414 (35, 49, 57, 58, 91, 123, 135, 171, 227, 229)	2.42%
	*tub2*	12/787 (30, 186, 539, 761, 767, 768, 769, 772, 776, 782, 783, 785, 787)	1.52%
*A.mediterranei*	ITS	1/539 (528)	0.19%
	LSU	1/320 (13)	0.31%
	*tub2*	28/796 (30, 186, 539, 761, 766, 767, 768, 769, 770, 771, 772, 773, 774, 775, 776, 777, 778, 779, 780, 781, 782, 783, 784, 785, 786, 787, 792, 794)	3.52%
*A.piptatheri*	ITS	10/528 (30, 38, 142, 177, 182, 213, 420, 421, 430, 431)	1.89%
	LSU	4/827 (417, 431, 480, 632)	0.48%
*A.pseudomarii*	ITS	5/556 (425, 528, 541, 560, 561)	0.90%
	*tef1*	7/416 (33, 38, 94, 173, 177, 212, 258)	1.68%
	*tub2*	1/718 (520)	0.14%

## ﻿Discussion

*Apiospora* has been revised using different approaches and its taxonomy and classification have changed several times since its introduction. The taxonomic classification of the genus in relation to *Arthrinium* has been a topic of debate (Crous and Groenewald 2013; Pintos and Alvarado 2021). Morphologically, *Apiospora* and *Arthrinium* share similarities in basauxic conidiogenesis. The conidia of *Apiospora* are typically lenticular or obovoid in the side view, with colours ranging from pale brown to brown. Conversely, the conidia of *Arthrinium* exhibit various shapes, such as angular, curved, fusiform, globose, navicular and polygonal (Kunze 1817; Hyde et al. 1998; Wang et al. 2018; Pintos and Alvarado 2021).

Recently, several revisions have been made in the course of unitary nomenclature resulting in the discovery of a plethora of new species, based on multigene phylogenies (Kwon et al. 2021; Pintos and Alvarado 2021, 2022; Liu et al. 2023). Currently there are 93 accepted species in *Apiospora* (Table 2), which are found on a wide range of materials.

In this study, *A.arundinis* and *A.lophatheri* were collected from the tropical region of Xishuangbanna City, while *A.coryli* was discovered in Ankang City and *A.oenotherae* was found in Lincang City, which are both subtropical regions. Consistent with previous studies, the majority of *Apiospora* species inhabit a diverse range of habitats primarily located in tropical and subtropical regions (Pintos and Alvarado 2021).

Specimens of *Apiospora* were collected from the Qinling Mountains in Ankang City and, in addition to *A.coryli*, Jiang et al. reported species found including *A.qinlingense* and *A.neochinensis* (Jiang et al. 2018; Jiang et al. 2020). Amongst these species, *A.coryli* was found to have longer conidiogenous cells (2.6–10.6 × 2.1–5.8 µm) compared to *A.qinlingense* (1–2 × 2–3 µm) and *A.neochinensis* (1.5–6.5 × 1–3.5 µm) and much larger spores than *A.qinlingense* (4–18.4 × 6.2–12.5 µm vs. 5–8 × 5–8 µm) (Table 6). These morphological differences suggest that *A.coryli* is distinct from *A.qinlingense* and *A.neochinensis*. This distinction is also supported by phylogenetic analysis shown in Fig. 1 which revealed that these species are phylogenetically distant from each other. Different species have been discovered in this region over several years, indicating that variation in species may be linked to the timing of collection, host plants, growth rates, developmental cycles and activity levels. These findings highlight the diversity of fungi within *Apiospora* genus in the subtropical region of the Qinling Mountains and suggest the existence of numerous undiscovered species with significant research potential. Further investigation is necessary to determine the value of specific regions for future research on fungi.

**Table 6.** Synopsis of new *Apiospora* species and species collected from the Qinling Mountains in *Apiospora*.

Species	Conidiogenous cells (µm)	Conidia (µm)	Host	Date	References
***Apiosporacoryli***	**2.6–10.6 × 2.1–5.8**	**4–18.4 × 6.2–12.5**	***Corylusyunnanensis***	**16 July 2021**	**Present study**
*A.qinlingense*	1–2	5–8	*Fargesiaqinlingensis*	27 June 2017	Jiang et al. 2018
*A.neochinensis*	1.5–6.5 × 1–3.5	8–12 × 5.5–9	*Fargesiaqinlingensis*	16 July 2018	Jiang et al. 2020

* Newly described taxa are in bold.

This paper reports the initial discovery of *A.lophatheri* on *Lophatherumgracile* (Poaceae). While numerous *Apiospora* have been discovered on Poaceae plants worldwide, previous research has primarily focused on bamboo, with limited investigation into herbaceous plants, such as *Lophatherum* (Liu et al. 2023). However, prior to this study, *Apiospora* had not been previously found on *Brunfelsia* (Solanaceae) and *Oenothera* (Onagraceae). While *Cercosporabrunfelsiicola* has been reported on other host *Brunfelsiauniflora* within the genus and *Pestalotiopsisoenotberae* has been identified specifically on *Oenotheralaciniata*, the restricted cultivation of these plants along with insufficient research on their associated fungi have resulted in few related studies (Venkatasubbaiah et al. 1991; Hidayat and Meeboon 2014). This discovery highlights potential interactions between these plant species and their fungal counterparts, emphasising the importance of uncommon herbaceous plants for fungal taxonomy alongside Rosaceae and silvicultural species like *Populus* (Peng et al. 2022; Lin et al. 2022). Hence, collecting various specimens is crucial for studying and identifying the fungi of *Apiospora*, while also promoting fungal diversity.

Most *Apiospora* species exhibit round or lenticular conidia, as demonstrated in this study. Nevertheless, the sizes of these conidia often overlap amongst morphologically similar, but phylogenetically distinct species within the genus *Apiospora*. For example, the conidia of *A.piptatheri* (7.5–10 × 7–9 µm) and *A.pseudosinense* (8–10 × 7–10 µm) are similar, but the two species are comparable despite their distinct evolutionary lineages in Fig. 1 (Crous and Groenewald 2013; Pintos et al. 2019). Therefore, relying merely on morphology can pose challenges for accurate identification.

The monophyly of taxonomic classification units at every rank is crucially important. Morphology is frequently insufficient for phylogenetic classification and, thus, molecular evidence has become increasingly significant and indispensable for identifying and classifying fungal taxa. In recent years, there has been a steady growth in DNA sequencing data available for *Apiospora* species (Crous and Groenewald 2013; Wang et al. 2018; Pintos et al. 2019), leading to the recognition of 93 species of *Apiospora*. Sequence data are accessible for ITS in 93 species, LSU in 80, *tef1* in 71 and *tub2* in 73, facilitating accurate and swift identification (Wang et al. 2018; Pintos et al. 2019). However, using ITS alone has its limitations in identifying *Apiospora* species. Therefore, LSU, *tef1*, *tub2* and multigene sequence data (ITS, LSU, *tef1* and *tub2*) have been particularly useful in establishing phylogenetic relationships and increasing accuracy in *Apiospora* identification. Furthermore, this study yielded 32 sequence datasets for four gene regions (ITS, LSU, *tef1* and *tub2*), enhancing our comprehension of the genus *Apiospora*. Novel species were identified by examining morphological and molecular characteristics, host associations and ecological distributions.

## Supplementary Material

XML Treatment for Apiospora arundinis

XML Treatment for Apiospora coryli

XML Treatment for Apiospora lophatheri

XML Treatment for Apiospora oenotherae

## ﻿Additional information

### Author ORCIDs

Shuji Li https://orcid.org/0009-0006-4734-8399

Chengming Tian https://orcid.org/0000-0002-3352-7664

### Data availability

All of the data that support the findings of this study are available in the main text or Supplementary Information.

## Supplementary materials

Supplementary material 1 Isolates and GenBank accession numbers used in the phylogenetic analyses Data type docx https://binary.pensoft.net/file/924328This dataset is made available under the Open Database License (http://opendatacommons.org/licenses/odbl/1.0/). The Open Database License (ODbL) is a license agreement intended to allow users to freely share, modify, and use this Dataset while maintaining this same freedom for others, provided that the original source and author(s) are credited.Shuji Li, Cheng Peng, Rong Yuan, Chengming Tian
