# Supplementary material for: ﻿Morphological and phylogenetic analyses reveal three new species of Apiospora in China
Source: MycoKeys. 2023 Oct 20;99:297–317. doi: 10.3897/mycokeys.99.108384 (PMC10612133; doi:10.3897/mycokeys.99.108384)
Supplement: Supplementary material 1 — Isolates and GenBank accession numbers used in the phylogenetic analyses [file mycokeys-99-297-s001.docx]

| **Species** | **Isolate/Strain** | **Host/ Substrate** | **Origin** | **GenBank accession numbers** | | | |
| --- | --- | --- | --- | --- | --- | --- | --- |
|  |  |  |  | **ITS** | **LSU** | ***tef1*** | ***tub2*** |
| *Apiospora acutiapica* | KUMCC 20-0210 (Type) | *Bambusa bambos* | China | MT946343 | MT946339 | MT947360 | MT947366 |
| *A. agari* | KUC 21333 (Type) | *Agarum cribrosum* | Korea | MH498520 | MH498440 | MH544663 | MH498478 |
| *A. aquatica* | MFLU 18-1628 (Type) | Submerged wood | China | MK828608 | MK835806 | NA | NA |
| *A. arctoscopi* | KUC 21331 (Type) | Egg of Arctoscopus japonicus | Korea | MH498529 | MH498449 | MN868918 | MH498487 |
| *A. arundinis* | CBS 10612 | Unkown substrate | Germany | KF144883 | KF144927 | KF145015 | KF144973 |
|  | LX 1918 | *Saccharum officinarum* | China | MW534386 | NA | MW584370 | MZ090019 |
| *A. aurea* | CBS 24483 (Type) | Air | Spain | AB220251 | KF144935 | KF145023 | KF144981 |
| *A. balearica* | CBS 145129 (Type) | Poaceae | Spain | MK014869 | MK014836 | MK017946 | MK017975 |
| *A. bambusae* | ICPM 6889 (Type) | Bamboo | China | MK014874 | MK014841 | MK017951 | MK017980 |
| *A. bambusicola* | MFLUCC 20-0144 (Type) | *Schizostachyum brachycladum* | Thailand | MW173030 | MW173087 | MW183262 |  |
| *A. biserialis* | CGMCC 320135 (Type) | Bamboo |  | MW481708 | MW478885 | MW522938 | MW522955 |
| ***A. brunfelsiae*** | **CFCC 58977 (Type)** | ***Brunfelsia brasiliensis*** | **China** | **OR125562** | **OR133584** | **OR139968** | **OR139976** |
|  | **LS 107** | ***Brunfelsia brasiliensis*** | **China** | **OR125563** | **OR133585** | **OR139969** | **OR139977** |
| *A. camelliae-sinensis* | LC 5007 (Type) | *Camellia sinensis* | China | KY494704 | KY494780 | KY705103 | KY705173 |
| *A. chromolaenae* | MFLUCC 17-1505 (Type) | *Chromolaena odorata* | Thailand | MT214342 | MT214436 | MT235802 | NA |
| *A. chiangraiense* | MFLUCC 21-0053 (Type) | Bamboo | Thailand | MZ542520 | MZ542524 | NA | MZ546409 |
| *A. cordylinae* | GUCC 10027 (Type) | Cordyline fruticosa | China | MT040106 | NA | MT040127 | MT040148 |
| ***A. coryli*** | **CFCC 58978 (Type)** | ***Corylus yunnanensis*** | **China** | **OR125564** | **OR133586** | **OR139974** | **OR139978** |
|  | **CFCC 58979** | ***Corylus yunnanensis*** | **China** | **OR125565** | **OR133587** | **OR139975** | **OR139979** |
| *A. cyclobalanopsidis* | CGMCC 320136 (Type) | *Cyclobalanopsidis glauca* | China | MW481713 | MW478892 | MW522945 | MW522962 |
| *A. descalsii* | CBS 145130 (Type) | *Ampelodesmos mauritanicus* | Spain | MK014870 | MK014837 | MK017947 | MK017976 |
| *A. dichotomanthi* | LC 4950 (Type) | *Dichotomanthus tristaniaecarpa* | China | KY494697 | KY494773 | KY705096 | KY705167 |
| *A. dongyingensis* | SAUCC 0302 (Type) | Bamboo | China | OP563375 | OP572424 | OP573264 | OP573270 |
| *A. esporlensis* | CBS 145136 (Type) | *Phyllostachys aurea* | Spain | MK014878 | MK014845 | MK017954 | MK017983 |
| *A. euphorbiae* | IMI 285638b | Bambusa | Bangladesh | AB220241 | AB220335 | NA | AB220288 |
| *A. fermenti* | KUC21289 (Type) | Seaweed | Korea | MF615226 | MF615213 | MH544667 | MF615231 |
| *A. gaoyouense* | CFCC 52301 (Type) | *Phragmites australis* | China | MH197124 | NA | MH236793 | MH236789 |
| *A. garethjonesii* | JHB004 (Type) | Bamboo | China | KY356086 | KY356091 | NA | NA |
| *A. gelatinosa* | HKAS 111962 (Type) | Bamboo | China | MW481706 | MW478888 | MW522941 | MW522958 |
| *A. guiyangensis* | HKAS 102403 (Type) | Poaceae | China | MW240647 | MW240577 | MW759535 | MW775604 |
| *A. guizhouensis* | LC 5322 (Type) | Air in karst cave | China | KY494709 | KY494785 | KY705108 | KY705178 |
| *A. hainanensis* | SAUCC 1681 (Type) | Bamboo | China | OP563373 | OP572422 | OP573262 | OP573268 |
| *A. hispanicum* | IMI 326877 (Type) | Maritime sand | Spain | AB220242 | AB220336 | NA | AB220289 |
| *A. hydei* | CBS 114990 (Type) | Bambusa tuldoides | China | KF144890 | KF144936 | KF145024 | KF144982 |
| *A. hyphopodii* | MFLUCC 15-0003 (Type) | Bamboo | China | KR069110 | NA | NA | NA |
| *A. ibericum* | AP 10118 (Type) | *Arundo donax* | Portugal | MK014879 | MK014846 | MK017955 | MK017984 |
| *A. intestini* | CBS 135835 (Type) | Gut of grasshopper | India | KR011352 | MH877577 | KR011351 | KR011350 |
| *A. italicum* | CBS 145138 (Type) | *Arundo donax* | Italy | MK014880 | MK014847 | MK017956 | MK017985 |
| *A. jatrophae* | CBS 134262 (Type) | *Jatropha podagrica* | India | JQ246355 | NA | NA | NA |
| *A. jiangxiensis* | LC 4577 (Type) | Maesa sp. | China | KY494693 | KY494769 | KY705092 | KY705163 |
| *A. kogelbergensis* | CBS 113333 (Type) | Restionaceae | South Africa | KF144892 | KF144938 | KF145026 | KF144984 |
| *A. koreanum* | KUC 21332 (Type) | Egg of Arctoscopus japonicus | Korea | MH498524 | MH498444 | MH544664 | MH498482 |
| *A. lageniformis* | KUC 21686 (Type) | Phyllostachys nigra | Korea | ON764020 | ON787759 | ON806624 | ON806634 |
| *A. locuta-pollinis* | LC 11683 (Type) | *Brassica campestris* | China | MF939595 | NA | MF939616 | MF939622 |
| *A. longistroma* | MFLUCC 11-0481 (Type) | Bamboo | Thailand | KU940141 | KU863129 | NA | NA |
| ***A. lophatheri*** | **CFCC 58975 (Type)** | ***Lophatherum gracile*** | **China** | **OR125566** | **OR133588** | **OR139970** | **OR139980** |
|  | **CFCC 58976** | ***Lophatherum gracile*** | **China** | **OR125567** | **OR133589** | **OR139971** | **OR139981** |
| *A. malaysiana* | CBS 102053 (Type) | Macaranga hullettii stem colonised by ants | Malaysia | KF144896 | KF144942 | KF145030 | KF144988 |
| *A. marianiae* | AP18219 (Type) | Phleum pratense | Spain | ON692406 | ON692422 | ON677180 | ON677186 |
| *A. marii* | CBS 49790 (Type) | Atmosphere, pharmaceutical excipients, home dust and beach sands | Spain | MH873913 | KF144947 | KF145035 | KF144993 |
| *A. marinum* | KU 21328 (Type) | Seaweed | China | MH498538 | MH498458 | MH544669 | MH498496 |
| *A. mediterranea* | IMI 326875 (Type) | Air | Spain | AB220243 | AB220337 | NA | AB220290 |
| *A. minutisporum* | 17E-042 (Type) | Soil | Korea | LC517882 | NA | LC518889 | LC518888 |
| *A. montagnei* | AP 301120 (Type) | *Arundo micrantha* | Spain | ON692408 | ON692424 | ON677182 | ON67718 |
| *A. mori* | MFLU 18-2514 (Type) | Morus australis | China | MW114313 | MW114393 | NA | NA |
| *A. mukdahanensis* | MFLUCC 22-0056 (Type) | Bambusoideae | Thailand | OP377735 | OP377742 | OP381089 | NA |
| *A. multiloculata* | MFLUCC 21-0023 (Type) | Bambusae | Thailand | OL873137 | OL873138 | NA | OL874718 |
| *A. mytilomorpha* | DAOM 214595 (Type) | Andropogon | India | KY494685 | NA | NA | NA |
| *A. neobambusae* | LC 7106 (Type) | Bamboo | China | KY494718 | KY494794 | KY806204 | KY705186 |
| *A. neochinensis* | CFCC 53036 (Type) | *Fargesia qinlingensis* | China | MK819291 | NA | MK818545 | MK818547 |
| *A. neogarethjonesii* | HKAS 102408 (Type) | Bambusae | China | MK070897 | MK070898 | NA | NA |
| *A. neosubglobosa* | JHB007 (Type) | Bamboo | China | KY356090 | KY356095 | NA | NA |
| *A. obovatum* | LC4940 (Type) | Lithocarpus | China | KY494696 | KY494772 | KY705095 | KY705166 |
| ***A. oenotherae*** | **CFCC 58972 (Type)** | ***Oenothera biennis*** | **China** | **OR125568** | **OR133590** | **OR139972** | **OR139982** |
|  | **LS 395** | ***Oenothera biennis*** | **China** | **OR125569** | **OR133591** | **OR139973** | **OR139983** |
| *A. ovata* | CBS 115042 (Type) | *Arundinaria hindsii* | China | KF144903 | KF144950 | KF145037 | KF144995 |
| *A. paraphaeosperma* | MFLUCC13-0644 (Type) | Bambusa | Thailand | KX822128 | KX822124 | NA | NA |
| *A. phragmitis* | CBS 135458 (Type) | Phragmites australis | Italy | KF144909 | KF144956 | KF145043 | KF145001 |
| *A. phyllostachydis* | MFLUCC 18-1101 (Type) | *Phyllostachys heteroclada* | China | MK351842 | MH368077 | MK340918 | MK291949 |
| *A. piptatheri* | CBS 145149 (Type) | *Piptatherum miliaceum* | Spain | MK014893 | MK014860 | MK017969 | NA |
| *A. pseudomarii* | GUCC 10228 (Type) | Aristolochia debilis | China | MT040124 | NA | MT040145 | MT040166 |
| *A. pseudohyphopodii* | KUC 21680 (Type) | Phyllostachys pubescens | Korea | ON764026 | ON787765 | ON806630 | ON806640 |
| *A. pseudoparenchymaticum* | LC 7234 (Type) | Bamboo | China | KY494743 | KY494819 | KY705139 | KY705211 |
| *A. pseudorasikravindrae* | KUMCC 20-0208 (Type) | *Bambusa dolichoclada* | China | MT946344 | NA | MT947361 | MT947367 |
| *A. pseudosinensis* | CBS 135459 (Type) | Bamboo | Netherlands | KF144910 | KF144957 | KF145044 | NA |
| *A. pseudospegazzinii* | CBS 102052 (Type) | Macaranga hullettii | Malaysia | KF144911 | KF144958 | KF145045 | KF145002 |
| *A. pterosperma* | CPC 20193 (Type) | *Lepidosperma gladiatum* | Australia | KF144913 | KF144960 | KF145046 | KF145004 |
| *A. pusillisperma* | KUC 21321 (Type) | Seaweed | Korea | MH498533 | MH498453 | MN868930 | MH498491 |
| *A. qinlingense* | CFCC 52303 (Type) | *Fargesia qinlingensis* | China | MH197120 | NA | MH236795 | MH236791 |
| *A. rasikravindrae* | NFCCI 2144 (Type) | Soil in karst cave | China | JF326454 | NA | NA | NA |
| *A. sacchari* | CBS 21230 | *Phragmites australis* | Korea | KF144919 | KF144965 | KF145050 | KF145008 |
| *A. saccharicola* | CBS 19173 | Air | Netherlands | KF144920 | KF144966 | KF145051 | KF145009 |
| *A. sargassi* | KUC21228 (Type) | *Sargassum fulvellum* | Korea | KT207746 | KT207696 | MH544677 | KT207644 |
| *A. sasae* | CBS 146808 (Type) | Sasa veitchii | Netherlands | MW883402 | MW883797 | MW890104 | MW890120 |
| *A. septata* | CGMCC 320134 (Type) | Bamboo | China | MW481711 | MW478890 | MW522943 | MW522960 |
| *A. serenensis* | IMI 326869 (Type) | Food, pharmaceutical excipients, atmosphere and home dust | Spain | AB220250 | AB220344 | NA | AB220297 |
| *A. setariae* | CFCC 54041 (Type) | Setaria viridis | China | MT492004 | NA | NA | NA |
| *A. setostroma* | KUMCC 19-0217 (Type) | Bambusoideae | China | MN528012 | MN528011 | MN527357 | NA |
| *A. sichuanensis* | HKAS 107008 (Type) | Poaceae | China | MW240648 | MW240578 | MW759536 | MW775605 |
| *A. sorghi* | URM 93000 (Type) | *Sorghum bicolor* | Brazil | MK371706 | NA | NA | MK348526 |
| *A. sphaerosperma* | CBS114314 (Type) | Hordeum vulgare | Iran | KF144904 | KF144951 | KF145038 | KF144996 |
| *A. stipae* | CBS 146804 (Type) | Stipa gigantea | Spain | MW883403 | MW883798 | MW890082 | MW890121 |
| *A. subglobosa* | MFLUCC 11-0397 (Type) | Bamboo | Thailand | KR069112 | KR069113 | NA | NA |
| *A. subrosea* | LC7292 (Type) | Bamboo | China | KY494752 | KY494828 | KY705148 | KY705220 |
| *A. taeanensis* | KUC21322 (Type) | Seaweed | Korea | MH498515 | MH498435 | MH544662 | MH498473 |
| *A. thailandica* | MFLUCC 15-0202 (Type) | Rotten wood | China | KU940145 | KU863133 | NA | NA |
| *A. vietnamense* | IMI 99670 (Type) | *Citrus sinensis* | Vietnam | KX986096 | KX986111 | NA | KY019466 |
| *A. xenocordella* | CBS 47886 (Type) | Soil from roadway | Zimbabwe | KF144925 | KF144970 | KF145055 | KF145013 |
| *A. yunnana* | MFLUCC 15-0002 (Type) | Bamboo | China | KU940147 | KU863135 | NA | NA |
| *Arthrinium crenatum* | CBS 146353B (Type) | Grass | France | MW208931 | MW208861 | MW221917 | MW221923 |
